# Distinguishing Proteins From Arbitrary Amino Acid Sequences

**DOI:** 10.1038/srep07972

**Published:** 2015-01-22

**Authors:** Stephen S.-T. Yau, Wei-Guang Mao, Max Benson, Rong Lucy He

**Affiliations:** 1 Department of Mathematical Sciences, Tsinghua University, Beijing, 100084, China; 2 Department of Computer Science, Seattle Pacific University, Seattle, WA 98119, USA; 3 Department of Biological Sciences, Chicago State University, Chicago, IL 60628, USA

## Abstract

What kinds of amino acid sequences could possibly be protein sequences? From all existing databases that we can find, known proteins are only a small fraction of all possible combinations of amino acids. Beginning with Sanger's first detailed determination of a protein sequence in 1952, previous studies have focused on describing the structure of existing protein sequences in order to construct the protein universe. No one, however, has developed a criteria for determining whether an arbitrary amino acid sequence can be a protein. Here we show that when the collection of arbitrary amino acid sequences is viewed in an appropriate geometric context, the protein sequences cluster together. This leads to a new computational test, described here, that has proved to be remarkably accurate at determining whether an arbitrary amino acid sequence can be a protein. Even more, if the results of this test indicate that the sequence can be a protein, and it is indeed a protein sequence, then its identity as a protein sequence is uniquely defined. We anticipate our computational test will be useful for those who are attempting to complete the job of discovering all proteins, or constructing the protein universe.

Structurally, proteins are sequences of amino acids^1^. Not all sequences of amino acids correspond to proteins, however. Previous studies^2^,^3^,^4^ have focused on describing the structure of existing protein sequences in order to construct the protein universe. Recent studies have considered the expansion of the protein universe and its relationship to the Big Bang^5^,^6^,^7^. So far, however, no study has developed a criteria for distinguishing a protein that is part of the protein universe from an amino acid sequence that is not a protein.

In this paper we will describe a 99.69% accurate computational test for determining if an arbitrary amino acid sequence can be a protein. Our computational test makes it possible to quickly determine whether a certain amino acid sequence can be a protein.

Our test is based on a geometric representation of the protein universe that we have constructed using the natural vector representation^8^. According to this approach, each protein sequence is represented by a unique point in coordinate space.

Our testing has shown that the points corresponding to proteins cluster together and it has revealed a fundamental limit on the distribution of each amino acid within proteins that was not evident previously.

## Results

The distribution of a specific amino acid “k” within an amino acid sequence can be described by three quantities:

- *n_k_* the number of occurrences of the amino acid “k” within the sequence.
- μ*_k_* the mean distance of the amino acid “k” from the first position.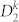 the second normalized central moment of the distribution of amino acid “k”.

Here is a more precise definition of the second and third quantities:

μ*_k_*: Let s[k][i] be the distance from the first position of the sequence to the location of the *i^th^* occurrence of the amino acid *k* and let 
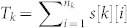
 be the total of the distances of all occurrences of *k* from the start position. Then μ*_k_* is simply *T_k_*/*n_k_*


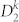
: Let n denote the length of the sequence, then 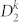 is defined by the following formula. 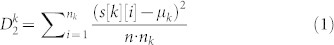


Using the standard abbreviations (A,R,N,D,C,E,Q,G,H,I,L,K,M,F,P,S,T,W,Y,V) for the 20 amino acids, each amino acid sequence can be represented by a point (called its *natural vector*) in 60-dimensional space with coordinates: 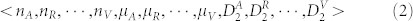


In general, the natural vector representation is not one to one. Many different amino acid sequences will correspond to the same point in 60-dimensional space. But that is not the case with actual protein sequences. The first key result of this paper is that after collecting all the known, reviewed complete protein sequences available in the *UniprotKB* database^9^ and computing their natural vectors, we verified that the natural vector representation is in fact one to one when it comes to known protein sequences.

We define *protein space* to be the set of all points in 60-dimensional space corresponding to protein sequences, and *amino acid space* to be the set of points that correspond to amino acid sequences with lengths ranging between the minimum and maximum lengths of the protein sequences.

**Figure 1:**
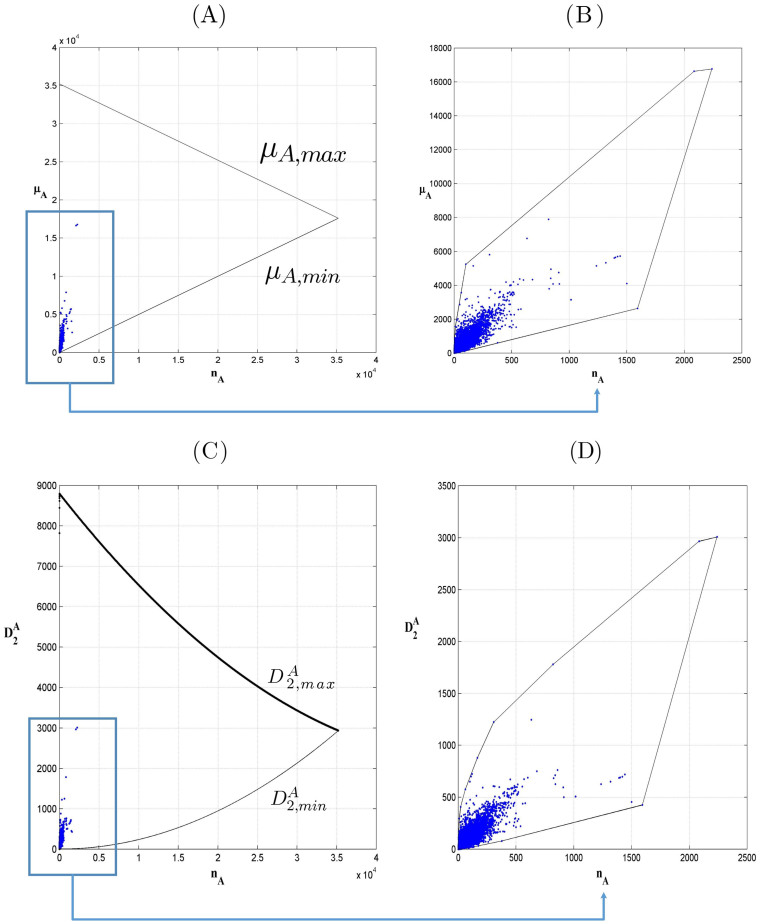
Alanine convex hull computed from the Uniprot 2013_03 dataset. Blue points in each of these four subfigures stand for vectors corresponding to proteins. (A) shows the picture in (*n_A_*, *μ_A_*) coordinate plane and (C) shows the picture 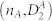 coordinate plane. (B) is the enlarged view of the protein area in (A). The black lines stand for the boundaries of the convex hull for protein area. (D) is the enlarged view of the protein area in (C). The black lines stand for the boundaries of the convex hull for the protein area.

In order to visualize how the points of protein space are distributed within amino acid space, we plotted them along two of the coordinate axes corresponding to the amino acid Alanine (A). Figures 1 (A) and (B) show the two dimensional projection onto the (*n_A_*, *μ_A_*) coordinate plane, while (C) and (D) show its projection onto the 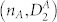 coordinate plane.

In the (*n_A_*, *μ_A_*) coordinate plane, the projection of amino acid space is bounded by the folowing three lines 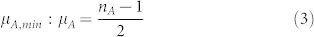

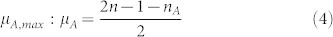

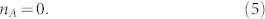


In the 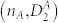 coordinate plane, the projection of the amino acid space is bounded by the following three curves 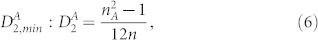

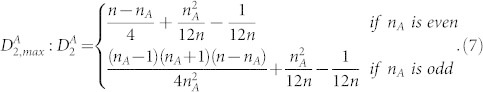

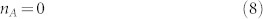


In these equations, n stands for the maximum length of proteins in the dataset. For the collection of known, reviewed complete protein sequences found in the *UniprotKB* data as of March 6, 2013, we found that n = 35213.

From Figure 1, we can clearly see that the points of protein space are clustered, rather than being broadly distributed. This makes us believe that as new protein sequences are described, their points will lie approximately within the *convex hull* of the points corresponding to known protein sequences.

Since there are no efficient algorithms to compute the convex hull in such a high dimensional space, we looked at three dimensional projections of the problem. For each amino acid “k”, we refer to the convex hull of points the form 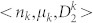 corresponding to protein sequences as the *“k”-protein area*. The *“k”-amino acid area* is the set of points corresponding to amino acid sequences with lengths in the range of actual protein sequences.

We performed several tests to check whether the boundaries of these protein areas will remain stable over time as more proteins are described. We computed the protein areas using the protein sequences listed in an earlier snapshot of the *UniprotKB* database. Then we tested to see whether more recently added protein sequences would lie within those convex hull. We found that nearly all of the new proteins sequences lie within the convex hulls, and the few that did not were found to lie very close to the convex hull boundaries.

Thus, a second key result of this paper is that we have found strong evidence for the validity of the following computational test:

  To check whether an arbitrary amino acid sequence can be a protein sequence, we start by computing its natural vector. Next we search a pre-computed database containing natural vectors that correspond to known protein sequences. If the natural vector is found within that database, we check whether the amino acid sequence is identical to the protein sequence we found. If they are identical, we have our answer. If they are not, the first key result of the paper allows us to conclude that the amino acid sequence is not a protein.

  If the natural vector of the amino acid sequence is not found in the database, we proceed to check whether each of the 20 points 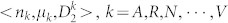 in 3-dimensional space lie within their corresponding “k”-protein area convex hull. If all these checks succeed, we conclude that this amino acid sequence could be a known or unknown protein. If on the other hand, not all points lie within the corresponding convex hulls, by this paper's second key result we would not expect the amino acid sequence to be a protein sequence.

## Methods

We took three snapshots of the *UniprotKB* database to test our hypotheses: *Uniprot 2013_03* (March 6, 2013)*, Uniprot 2014_03* (March 19, 2014), and *Uniprot 2014_06* (June 11, 2014). In each case, only the reviewed, complete proteins were selected.

We followed the same procedure for generating all three datasets. We added the keyword “Complete proteome[KW-0181]” to select only complete sequences, eliminating sequences with missing amino acids. We also added the keyword “Reviewed” to restrict to only reviewed proteins. Sequence redundancy was not reduced. After the download, the datasets were normalized by removing protein sequences containing Selenocysteine (U) and Pyrrolysine (O), as well as protein sequences containing placeholders (B, Z, J, X).

Only a small number of protein sequences were eliminated by this normalization process: The number of protein sequences containing Selenocysteine, Pyrrolysine or one of the four placeholders is 971 in the *Uniprot 2013_03* dataset, 1010 in the *Uniprot 2014_03* dataset, and 1044 in the *Uniprot 2014_06 dataset*. See Table 1 for details of the counts of sequences in the three snapshots of UniprotKB:

We used the *Uniprot 2013_03* and *Uniprot 2014_03* datasets to verify the first key result (the natural vector mapping produces a one-to-one correspondence between proteins and 60-dimensional natural vectors). As can be seen from Table 2, the number of distinct protein sequences in each of these datasets was equal to the number of distinct natural vectors.

We also performed several tests to check whether our computational test was effective for determining whether a given amino acid sequence could be a protein or not.

**Figure 2:**
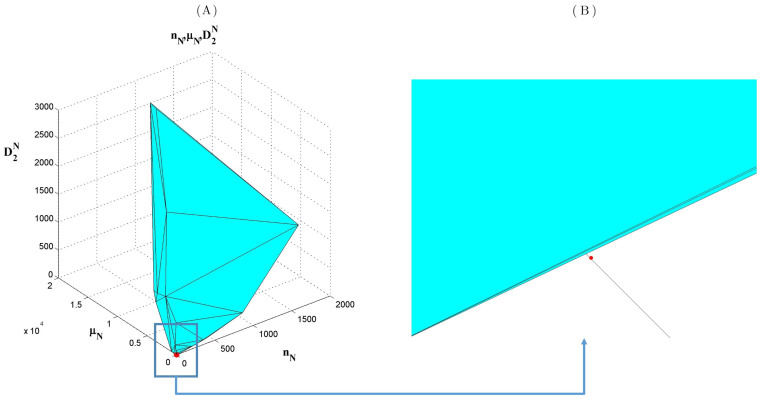
Protein sequence (Access ID P85817) lying outside the Asparagine convex hull. In the subfigure (A), the cyan surfaces stand for the surfaces of convex hulls in 3-dimensional space. The red point stands for the coordinate 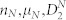 for this sequence. The subfigure (B) is an enlarged view of subfigure (A) showing that the point really falls outside the convex hull.

First, we used the 391704 distinct protein sequences contained in both *Uniprot 2013_03* and *Uniprot 2014_03* to construct twenty 3-dimensional convex hulls using the Qhull algorithm^10^. Then we checked to see how many of the 3810 sequences contained solely in *Uniprot 2014_03* failed to lie outside of the twenty convex hulls. We found that only 14 sequences (only 0.37% of the 3810 sequences) lie outside one of the convex hulls. We also used all 392455 sequences contained in *Uniprot 2013_03* to construct convex hulls and the results were the same. None of these 14 sequences were far from the boundaries of the convex hulls. In Table 3, we list the 14 sequences and their distances from the convex hulls, and in Figure 2, we give a graphical display of one of the 14 sequences lying outside one of the convex hulls.

Second, we computed new convex hulls using the above 391704 protein sequences plus the 14 sequences that failed the first test. We then checked each of the 5820 sequences that are contained in *Uniprot 2014_06* but not in the intersection of *Uniprot 2013_03 and Uniprot 2014_03* to see how many of them failed to lie within one of the convex hulls. The results of our testing showed that only 18 sequences (only 0.31% of the 5820 sequences) lie outside one of the convex hulls. As in the previous test, none of these 18 sequences were far from the boundaries of the convex hulls. See the supplementary information for tabulation of the distance of each sequence from the convex hulls. Table 4 lists the 18 sequences and their distance from their respective convex hulls.

As a final test, we tested to see whether the Top 7^11^, HOP2^12^, and GLUT1^13^ protein sequences lie within the convex hulls constructed from the sequence contained in both *Uniprot 2013_03* and *Uniprot 2014_03*. All three proteins lie inside the twenty convex hulls as expected.

## Discussion

Although there were a few proteins in each test which lay outside at least one of the convex hulls, our results are quite promising. The percentage of proteins failing the test were small in both cases (0.37% and 0.31%). In addition, proteins not contained within a convex hull were never far from the boundary of the convex hull. We have every indication to believe that as more protein sequence data becomes available the convex hulls we compute will become more and more reliable and eventually stabilize to give an accurate test for quickly determining whether a certain amino acid sequence can be a protein sequence.

In particular we envision this test being utilized by researchers in at least two different areas. Protein designers attempting to synthesize a new protein could use this test to quickly screen out amino acid sequences which are unlikely to be proteins before undertaking more expensive synthesis work in the laboratory. Similarly, biologists studying alternative splicing^14^,^15^ now have a new tool to predict whether an alternative splicing would produce a real protein sequence.

### Derivation for the equations of the boundaries of amino acid space

#### The boundaries of amino acid k in the (n_k_, μ_k_) plane

We need to find the minimum value μ_k,min_ and maximum value μ_k,max_ of the mean among all sequences of length *n* with *n_k_* occurrences of the amino acid *k*:

Theorem 1. Let n_k_ be the number of occurrences of amino acid *k* in a sequence of length *n*. Then
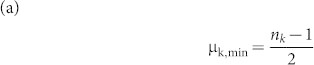

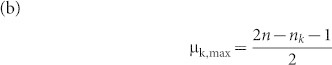


Proof: (a) Obviously if we choose the amino acid k to be distributed in positions 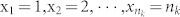, then we will get the minimum value of μ_k_ which is 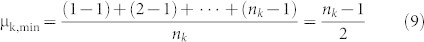


(b) Similarly if we choose the amino acid k to be distributed in positions 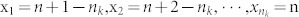, then we will get the maximum value of μ_k_ which is 
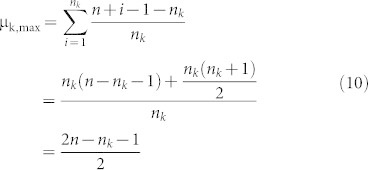



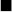
Remark: The line 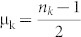 for 1 ≤ n_k_ ≤ *c* gives the lower boundary of the region while the line 
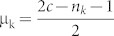
 for 1 ≤ n_k_ ≤ *c* gives the upper boundary of the region. Here c is the maximum length of the sequences in our data set.

#### The boundaries of amino acid k in the 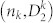 plane

We start by determining the maximum value 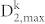 of the second normalized moment among all sequences of length *n* with *n_k_* occurrences of the amino acid *k*. The following lemmas show that any distribution which doesn't have amino acid *k* in both the first and last positions will have a second normalized moment which is not maximal.

Lemma 2. Let 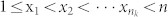 be a distribution of the amino acid *k*. Let 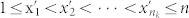 be another distribution with the properties that 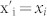 for 1 ≤ i ≤ n_k_ − 1 and 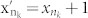. Then 
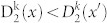

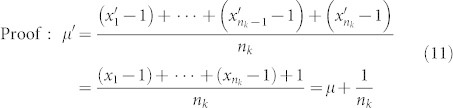

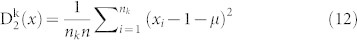

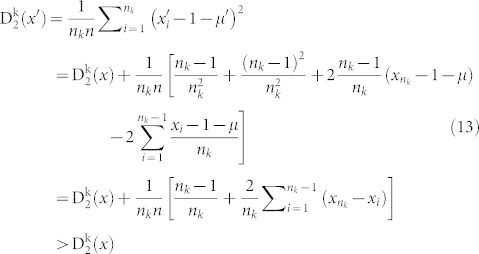



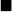
Lemma 3. let 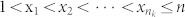 be a distribution of the amino acid *k*. Let 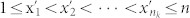 be another distribution with the properties that 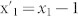 and 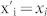 for 2 ≤ i ≤ n_k_. Then 
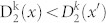


Proof: 
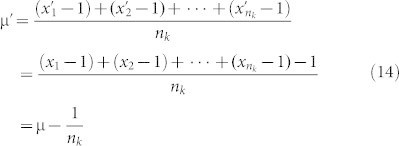

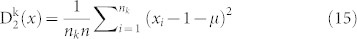

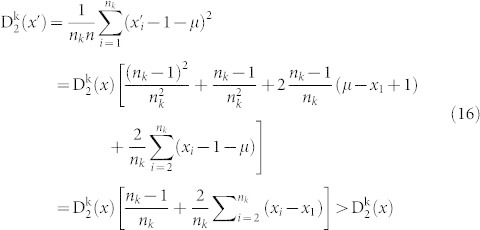



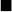
We can now determine the precise distribution of the amino acid *k* where the maximum value of the second normalized moment is assumed.

Theorem 4. Let 0 < n_k_ ≤ *n* be fixed positive integers. Let 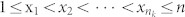 be a distribution of the amino acid k. If 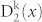 attains the maximum value 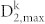 among all possible distributions, then 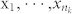 is of the following form:

Case 1 n_k_ even integer 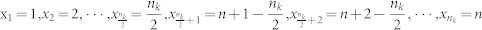


Case 2 n_k_ odd integer 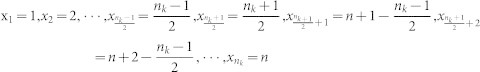


Proof: We shall only prove the case when n_k_ is an even integer since the proof for n_k_ an odd integer is the same. We use induction on n_k_. Let 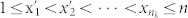 be any distribution of the amino acid k. In view of Lemma 2 and Lemma 3, we have 
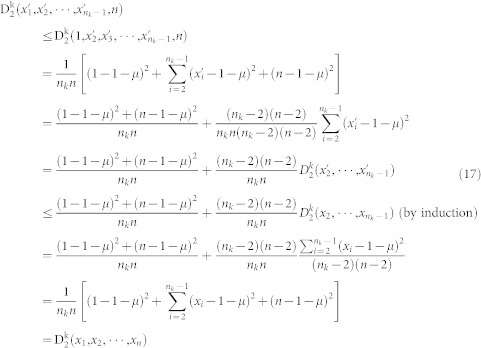



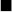
Now that the arrangement of the amino acids is known, the value of 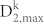 can be computed.

Theorem 5. Let 0 < n_k_ ≤ *n* be fixed positive integers. Then the maximum value 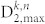 of all possible distributions 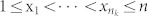 of amino acid k is given by the following formulas.

(1) n_k_ even, then 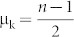

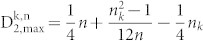


(2) n_k_ odd, then 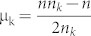

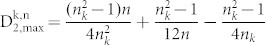


Proof (1) If n_k_ is even, in view of Theorem 4, we have 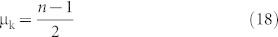

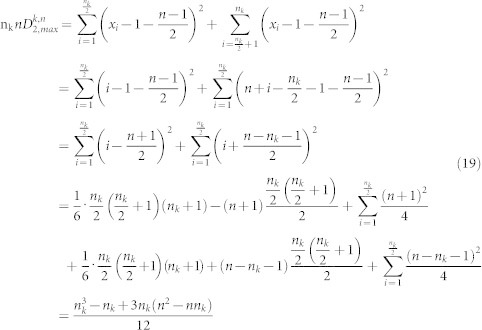

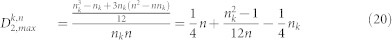


(2) If n_k_ is odd, then in view of Theorem 4 
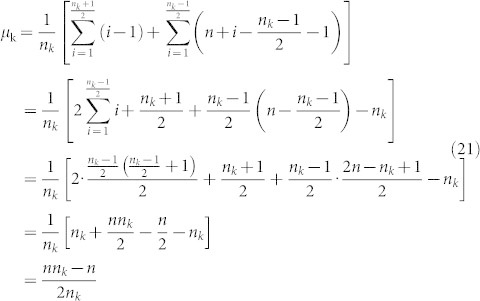

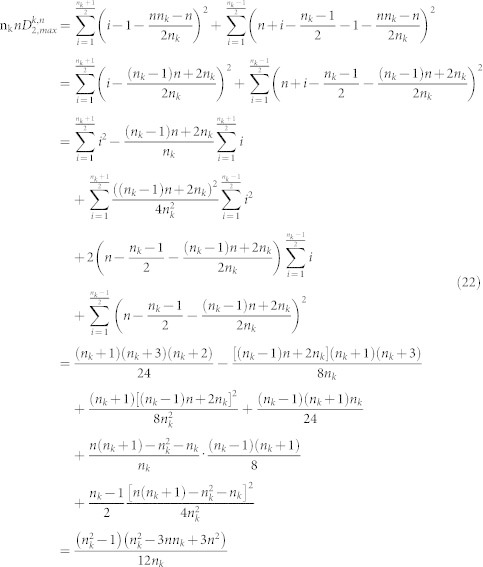

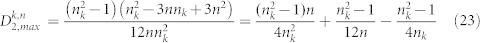



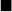
Corollary 6. Let c be the maximum length of the sequences in the dataset. Let n_k_, the number of occurrences of the amino acid k, be a fixed even integer. Then 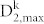 in the data set is given by 
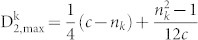


Proof: Let 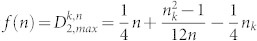 for n_k_ ≤ *n* ≤ *c*. We need to find the maximum value of *f*(*n*). It is easy to show that f(n) gets its minimum value at the point 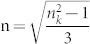. When n is larger than 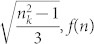 increases as n increases. Since 
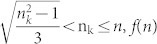
 gets its maximum value at point c.

Therefore, 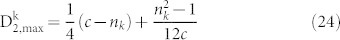



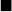
Corollary 7. Let c be the maximum length of the sequences in the dataset. Let n_k_, the number of occurrences of the amino acid k, be a fixed odd integer. Then 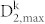 in the data set is given by 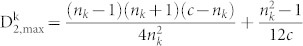


Proof: Let 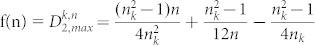 for n_k_ ≤ *n* ≤ *c*. In this case, the minimum value of *f*(*n*) is assumed at 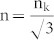. When n is larger than 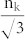., f(n) increases as n increases. Since 
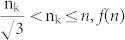
 gets its maximum value at point c.

Therefore, 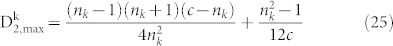



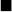
The final step is to compute the minimum value 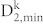 of the second normalized moment. This value is assumed when the amino acid *k* occupies the first n_k_ positions.

Proposition 8. Let 0 < n_k_ ≤ *n* be fixed positive integers. Let x_i_ = *i*, 1 ≤ i ≤ n_k_, be a distribution of the amino acid k, Then

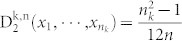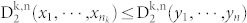 where 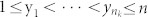 is any distribution of the amino acid k.

Proof: We only need to prove (1) since (2) is obvious. 
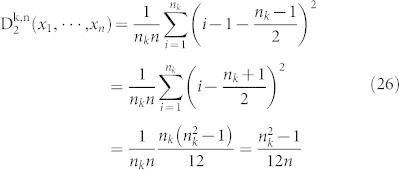



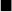
Corollary 9. Let c be the maximum length of the sequences in the dataset. Let n_k_, the number of occurrences of the amino acid k, be a fixed number. Then 
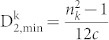


## Author Contributions

S.S.-T.Y. conceived the two criteria and designed the studies. W.G.M. carried out the data analysis including figures drawing. S.S.-T.Y., W.G.M. and R.L.H. provided the theory in Supplementary Materials. All authors participated in writing up the paper. The final version is done by M.B.

## Supplementary Material

Supplementary Information Supplementary Information

## Figures and Tables

**Table 1:** Detailed counts of sequences in the three snapshots of UniprotKB

Number of Distinct Sequences in *Uniprot 2013_03*	392455
Number of Distinct Sequences in *Uniprot 2014_04*	395514
Number of Distinct Sequences in *Uniprot 2014_06*	397348
Number of Distinct Sequences *Uniprot 2013_03 & Uniprot 2014_04*	391704
Number of Distinct Sequences Contained in all three	391528
Number of Sequences in *Uniprot 2013_03* but not *Uniprot 2014_03*	751
Number of Sequences in *Uniprot 2014_03* but not *Uniprot 2013_03*	3810
Number of Sequences in *Uniprot 2013_03* & *Uniprot 2014_03,* but not *Uniprot 2014_06*	176
Number of Sequences in *Uniprot 2014_06* but not in the intersection of *Uniprot 2013_03* & *Uniprot 2014_03*	5820

**Table 2:** Corresponding between sequence counts and natural vectors

	Number of Sequences Before Normalization	Number of Sequences	Number of Distinct Sequences	Number of Natural Vectors	Number of Distinct Natural Vectors
*Uniprot 2013_03*	472284	471313	392455	471313	392455
*Uniprot 2014_03*	475547	474537	395514	474537	395514

**Table 3:** The 14 protein sequence outliers in *Uniprot 2014_03* and their distances from the convex hulls

No.	Sequence Length	Access ID	Convex hull(s) the sequences fall outside	Distance from point to convex hull
1	11	P85817	Asparagine (N)	0.0177
2	16	P81071	Aspartic acid (D)	0.0110
3	19	P68116	Aspartic acid (D)	0.0018
4	20	P14469	Isoleucine (I)	0.003
5	199	Q9ZVZ9	Histidine (H)	0.0000208
6	211	P33191	Tyrosine (Y)	0.0027
7	237	Q6M923	Glutamine (Q)	0.0362
8	287	P50751	Proline (P)	0.0044
9	392	Q5A8I8	Proline (P)	33.9023
10	1086	Q59XL0	Methionine (M)	0.4508
11	1129	Q9QR71	Glutamic acid (E)	5.5955 (E)
			Glutamine (Q)	1.4455 (Q)
12	1404	Q59SG9	Serine (S)	0.2427
13	2346	A1Z8P9	Glycine (G)	0.2179
14	3461	P62288	Arginine (R)	1.8593

**Table 4:** The 18 protein sequence outliers from *Uniprot 2014_06* and their distances from the convex hulls

No.	Sequence Length	Access ID	Convex hull(s) the sequences fall outside	Distance from point to convex hull
1	20	P82867	Aspartic acid (D)	0.0045
2	19	P68214	Aspartic acid (D)	0.0309
3	15	P80612	Alanine (A)	0.3850
4	267	P14918	Arginine (R)	0.0000042949
5	150	P27787	Phenylalanine (F)	0.00025511
6	372	Q5AKU5	Histidine (H)	0.00013742
7	105	Q2RB28	Leucine (L)	0.000053612
8	105	B9GBM3	Leucine (L)	0.000053612
9	94	Q5G8Z3	Aspartic acid (D)	0.0022
10	391	P46525	Serine (S)	0.1044
11	838	P08489	Methionine (M)	0.0840
			Valine (V)	3.0627
12	848	P10388	Glutamic acid (E)	0.1269
			Methionine (M)	0.1408
			Valine (V)	3.1465
13	240	P04702	Glycine (G)	0.000036147
14	240	P06677	Glycine (G)	0.000036147
15	240	P04703	Glycine (G)	0.000036147
16	240	P06676	Glycine (G)	0.000036147
17	267	P04698	Glycine (G)	0.000070559
18	187	B6U769	Proline (P)	0.0037
