# Supplementary material for: Distinguishing Proteins From Arbitrary Amino Acid Sequences
Source: Sci Rep. 2015 Jan 22;5:7972. doi: 10.1038/srep07972 (PMC4302309; doi:10.1038/srep07972)
Supplement: Supplementary Information [file srep07972-s1.pdf]

Distinguishing Proteins From Arbitrary Amino Acid Sequences

Authors: Stephen S.-T. Yau[1*], Wei-Guang Mao[1**], Max Benson[2], Rong Lucy He[3]

Affiliations:
[1] Department of Mathematical Sciences, Tsinghua University, Beijing, 100084, China.
[2] Department of Computer Science, Seattle Pacific University, Seattle, WA 98119, USA.
[3]Department of Biological Sciences, Chicago State University, Chicago, IL 60628, USA.
[*]To whom correspondence should be addressed. Email: yau@uic.edu (SSTY).
[**]Co-first author

Supplementary Information:

Databases.
You can get the datasets we used via the links below:
Uniprot release 2013_03_Complete proteome_Reviewed_Normalized:
http://r720.math.tsinghua.edu.cn/Data/proteome/Uniprot release 2013_03_Complete proteome_Reviewed_Normalized.fasta

Uniprot release 2014_03_Complete proteome_Reviewed_Normalized:
http://r720.math.tsinghua.edu.cn/Data/proteome/Uniprot release 2014_03_Complete proteome_Reviewed_Normalized.fasta

Uniprot release 2014_06_Complete proteome_Reviewed_Normalized.fasta
http://r720.math.tsinghua.edu.cn/Data/proteome/Uniprot release 2014_06_Complete proteome_Reviewed_Normalized.fasta

Qhull Algorithm. We used the convhulln() function supplied with MATLAB to compute all convex hulls. This function is based on Qhull algorithm. We used a publicly available MATLAB function called inhull() to test whether or not a given point was in the convex hull. The code for this function can be accessed through the link: http://www.mathworks.com/matlabcentral/fileexchange/10226-inhull

Additional Figures. The following figures show the protein areas for each of the twenty amino acids. Figure 1 below shows the same information as Figure 1 in the main text, while the other figures show the protein areas for the other nineteen amino acids. The figure were generated these figures as described in the main text, using the dataset *Uniprot 2013_03*. Blue points in each of these four subfigures stand for natural vectors corresponding to proteins. From left to right and top to bottom, the four subfigures are named (A), (B), (C) and (D). (A) shows the picture in $(\boldsymbol{n_k}, \boldsymbol{\mu_k})$ coordinate and (C) shows the picture $(\boldsymbol{n_k}, \boldsymbol{D_2^k})$ coordinate. (B) is the enlarged view of the protein area in (A). The black lines stand for the boundaries of the convex hull for protein area. (D) is the enlarged view of the protein area in (C). The black lines stand for the boundaries of the convex hull for the

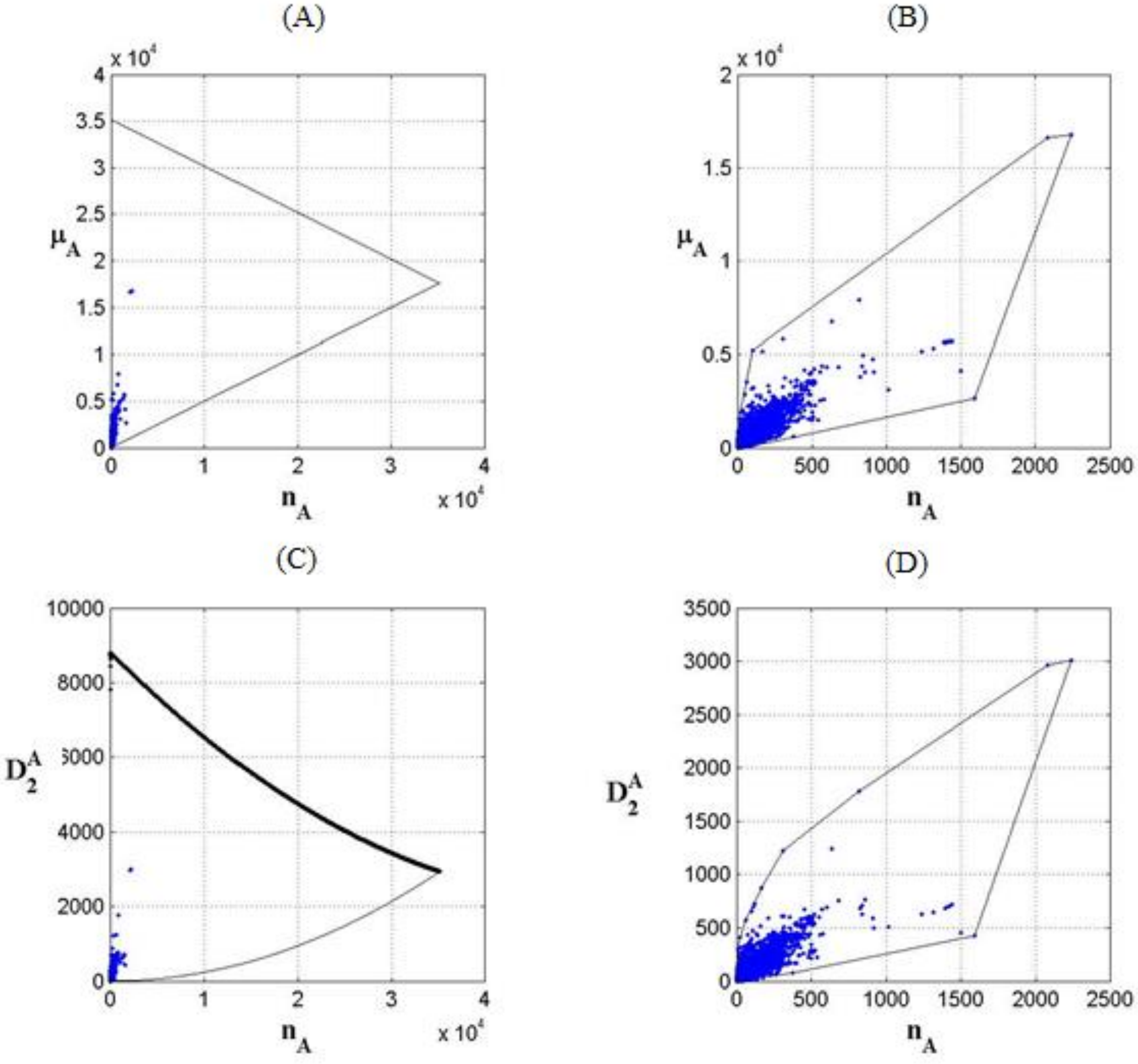

Figure 1: Protein Area Detail for Alanine

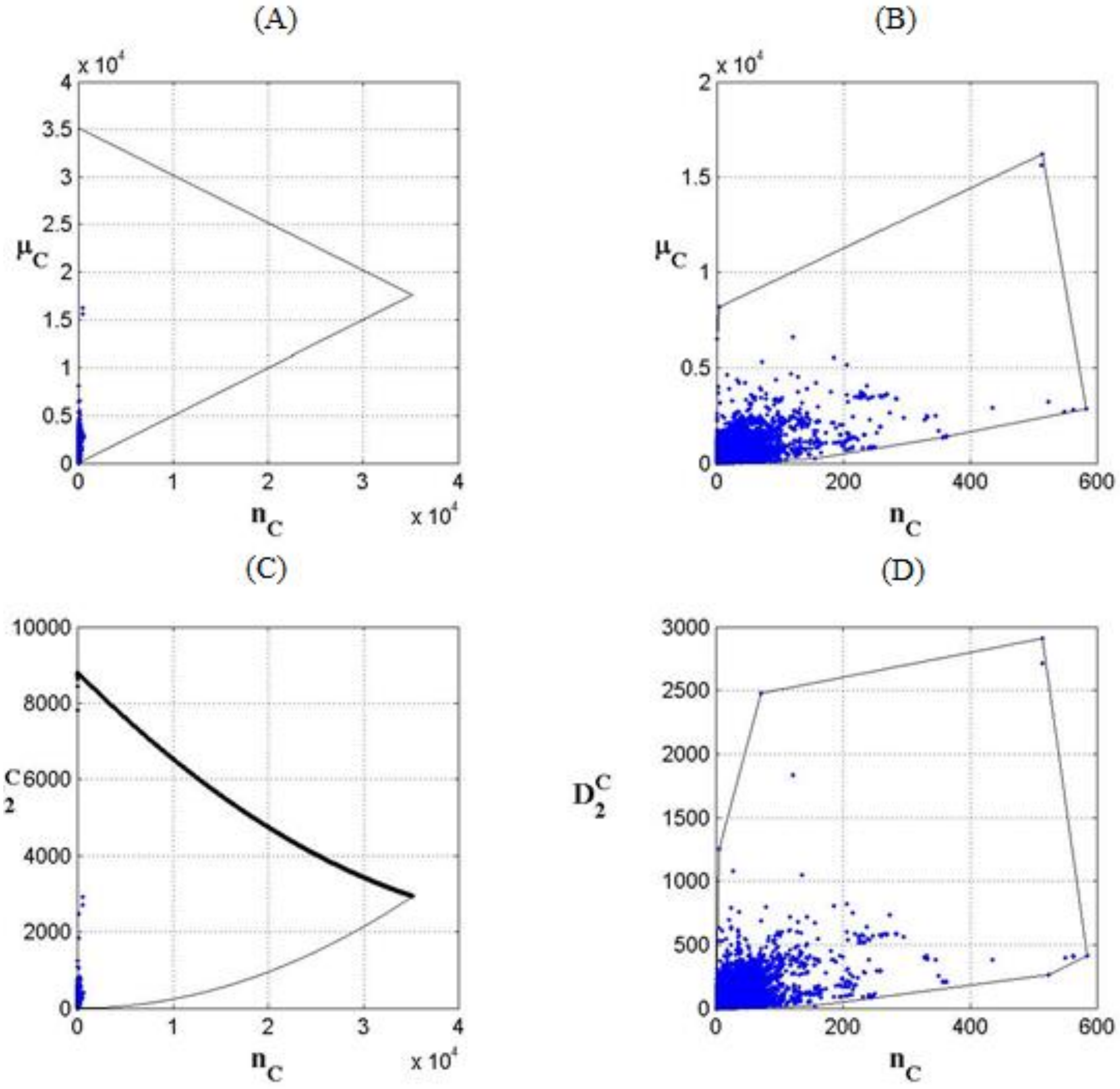

Figure 2: Protein Area Detail for Cysteine

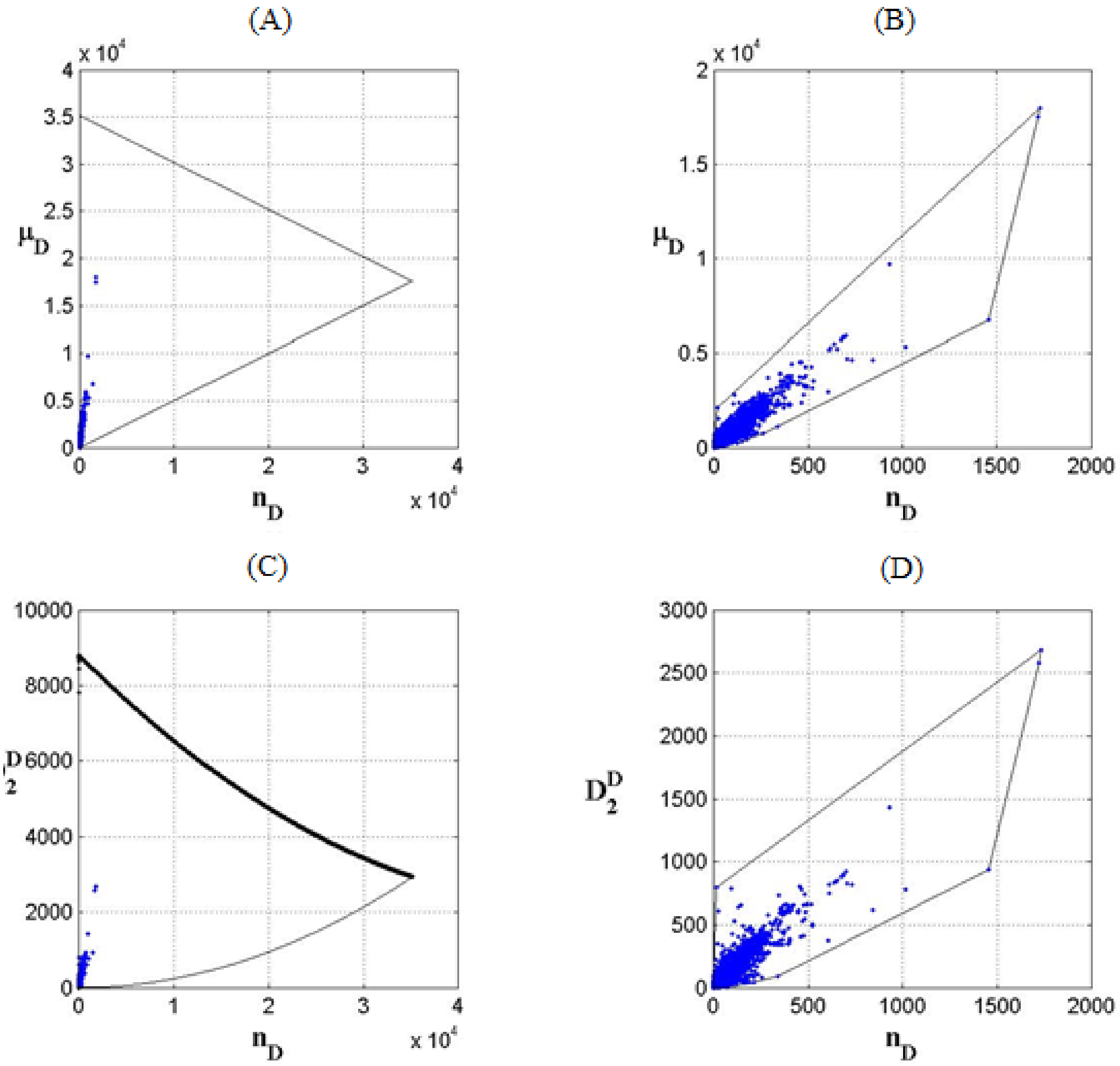

Figure 3: Protein Area Detail for Aspartic Acid

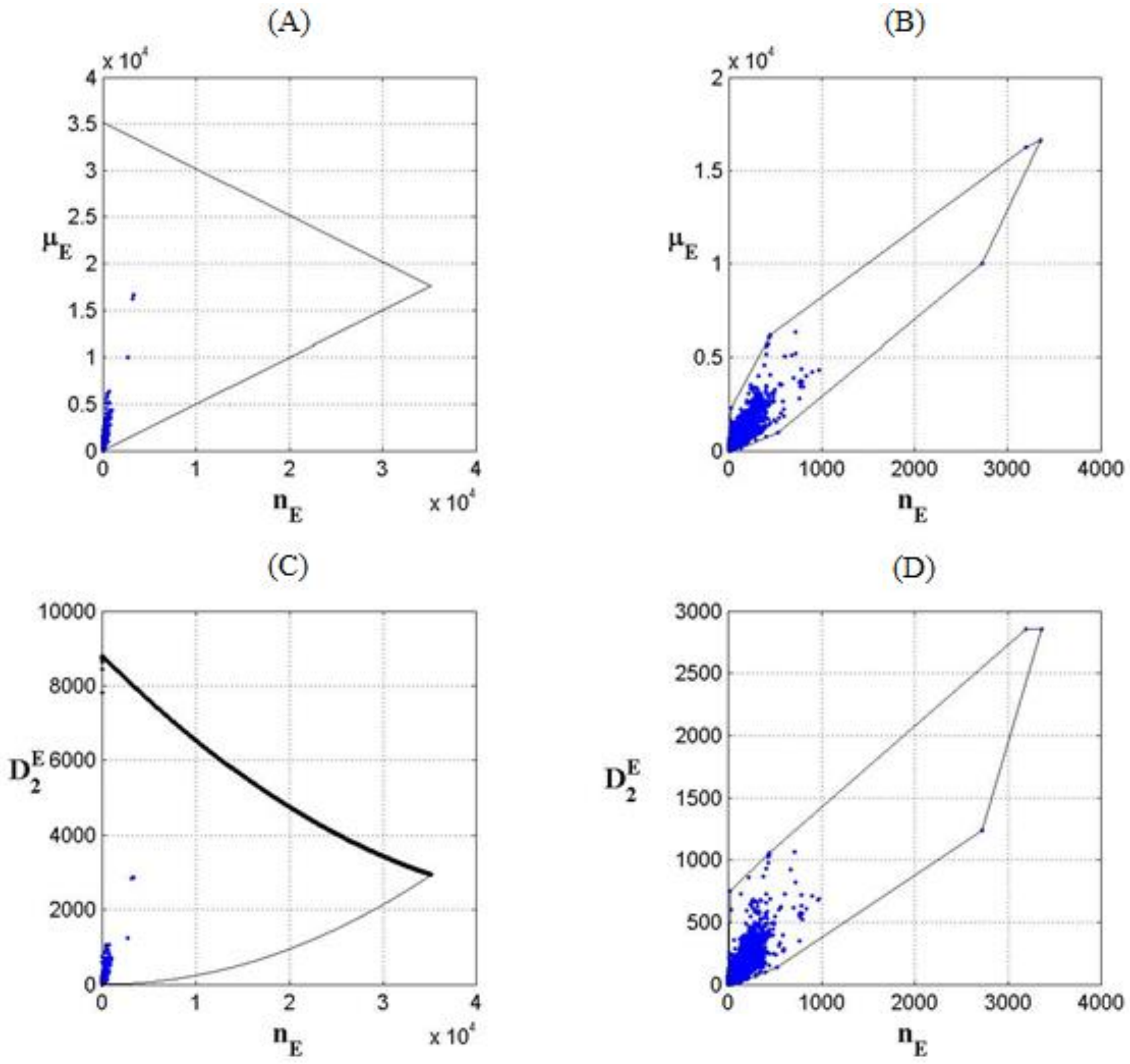

Figure 4: Protein Area Detail for Glutamic Acid

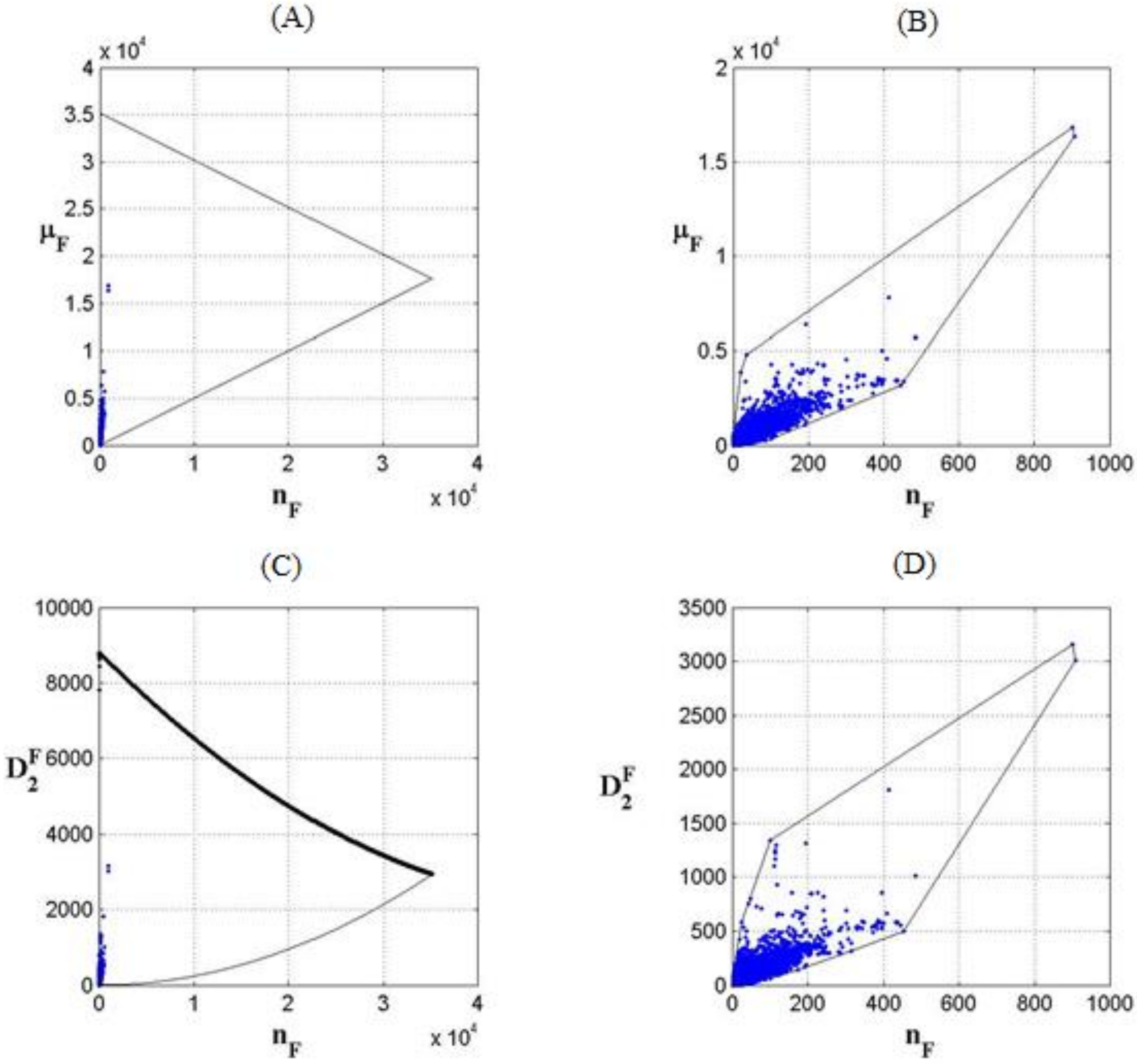

Figure 5: Protein Area Detail for Phenylalanine

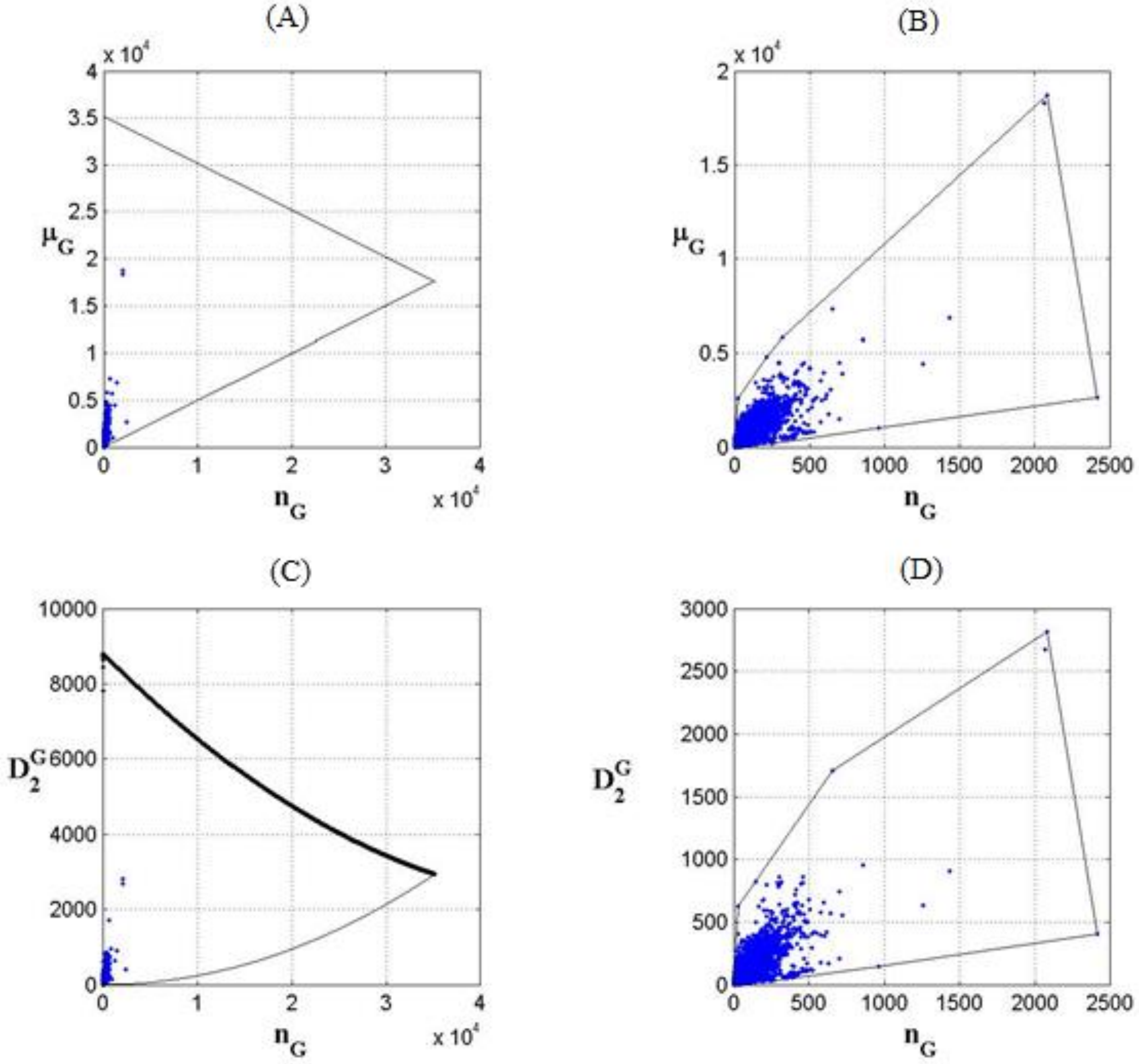

Figure 6: Protein Area Detail for Glycine

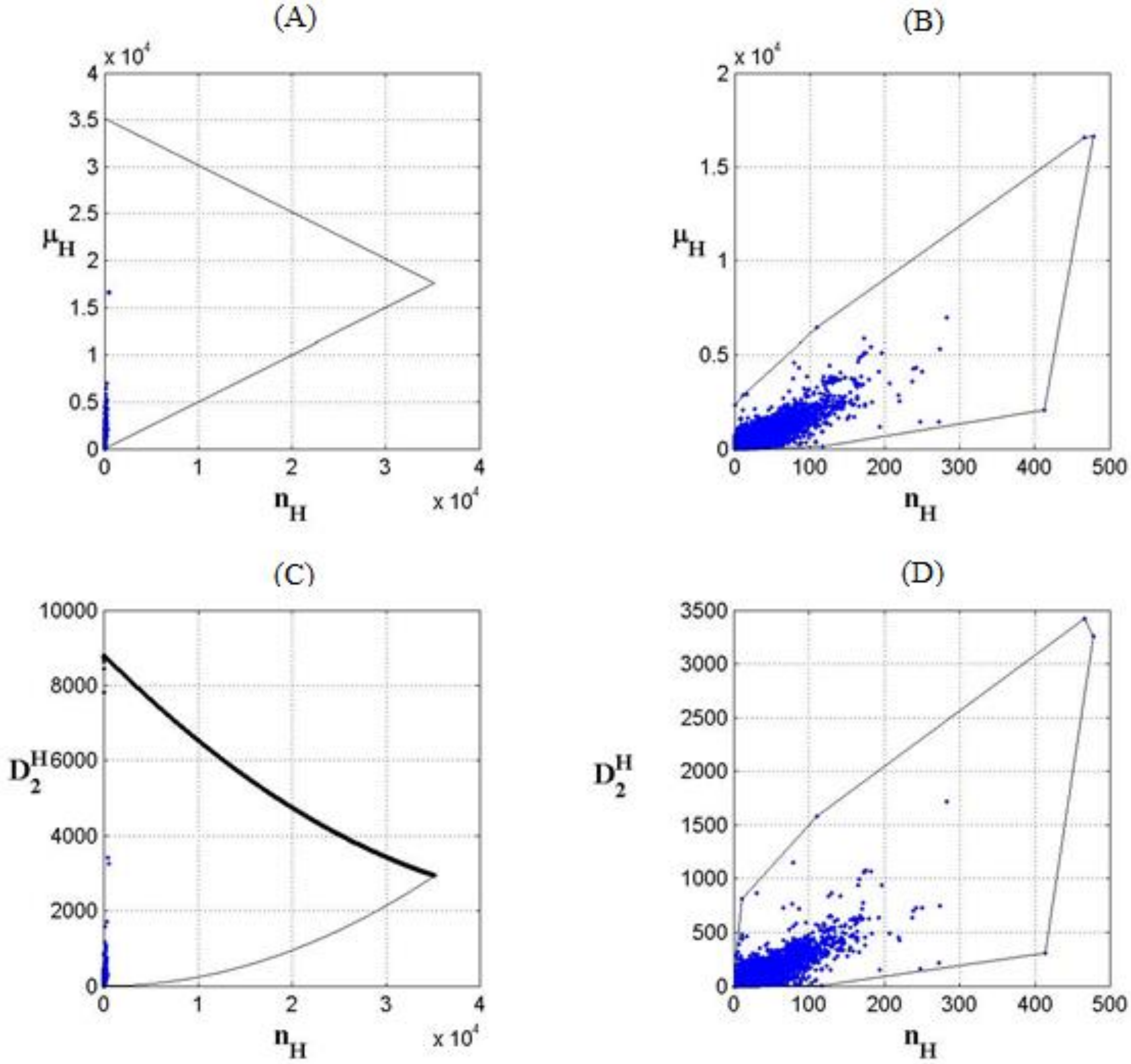

Figure 7: Protein Area Detail for Histidine

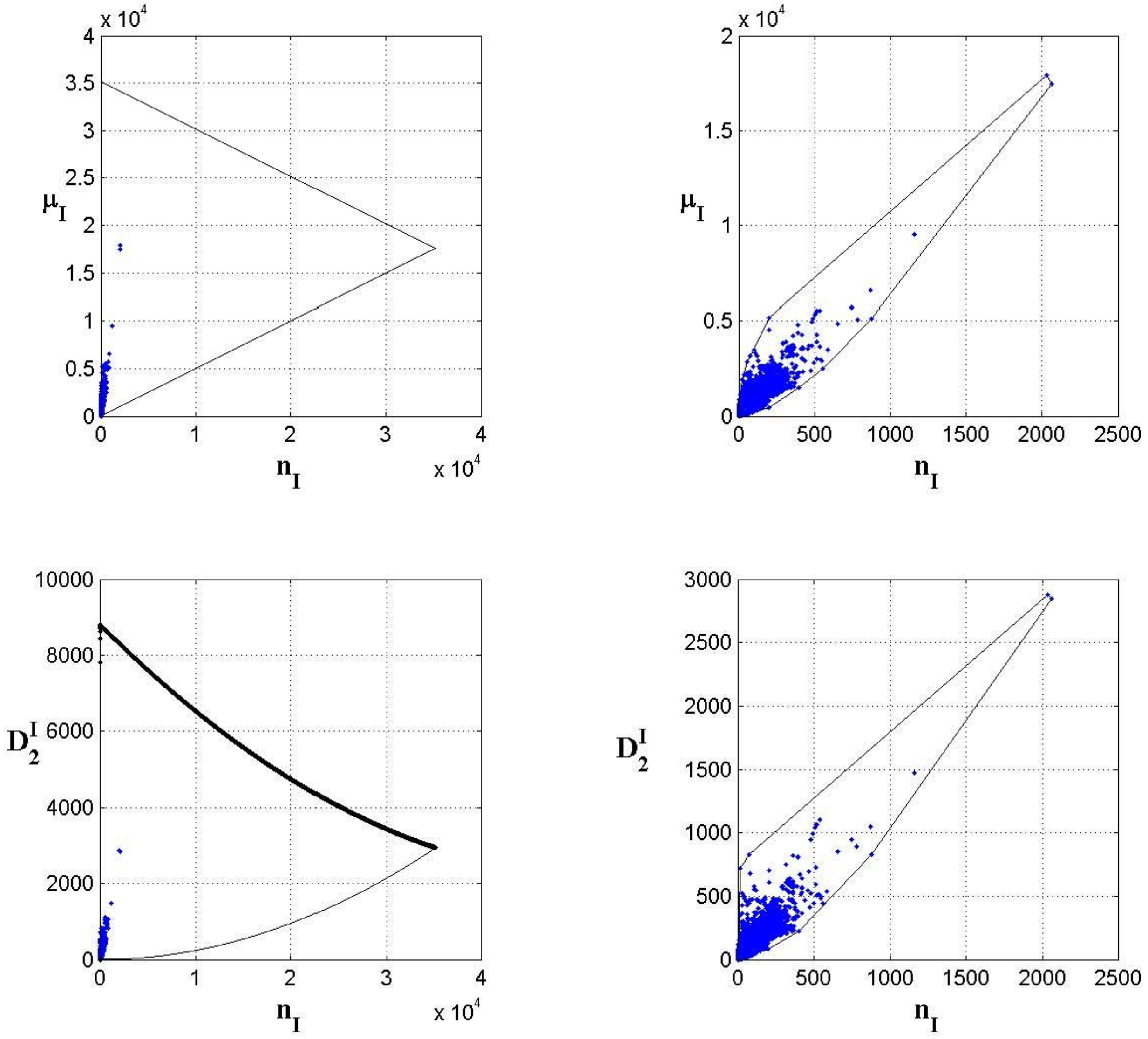

Figure 8: Protein Area Detail for Isoleucine

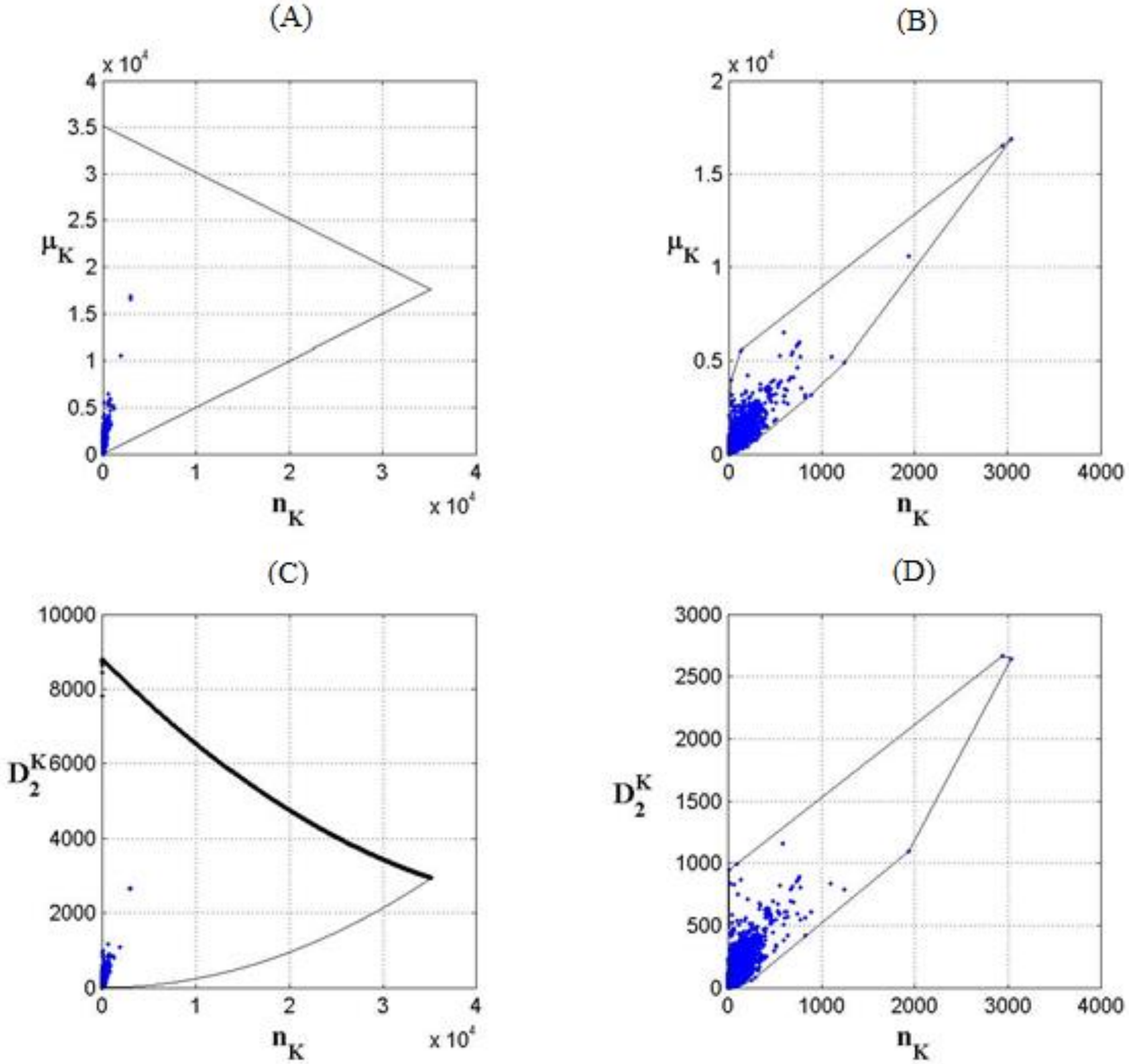

Figure 9: Protein Area Detail for Lysine

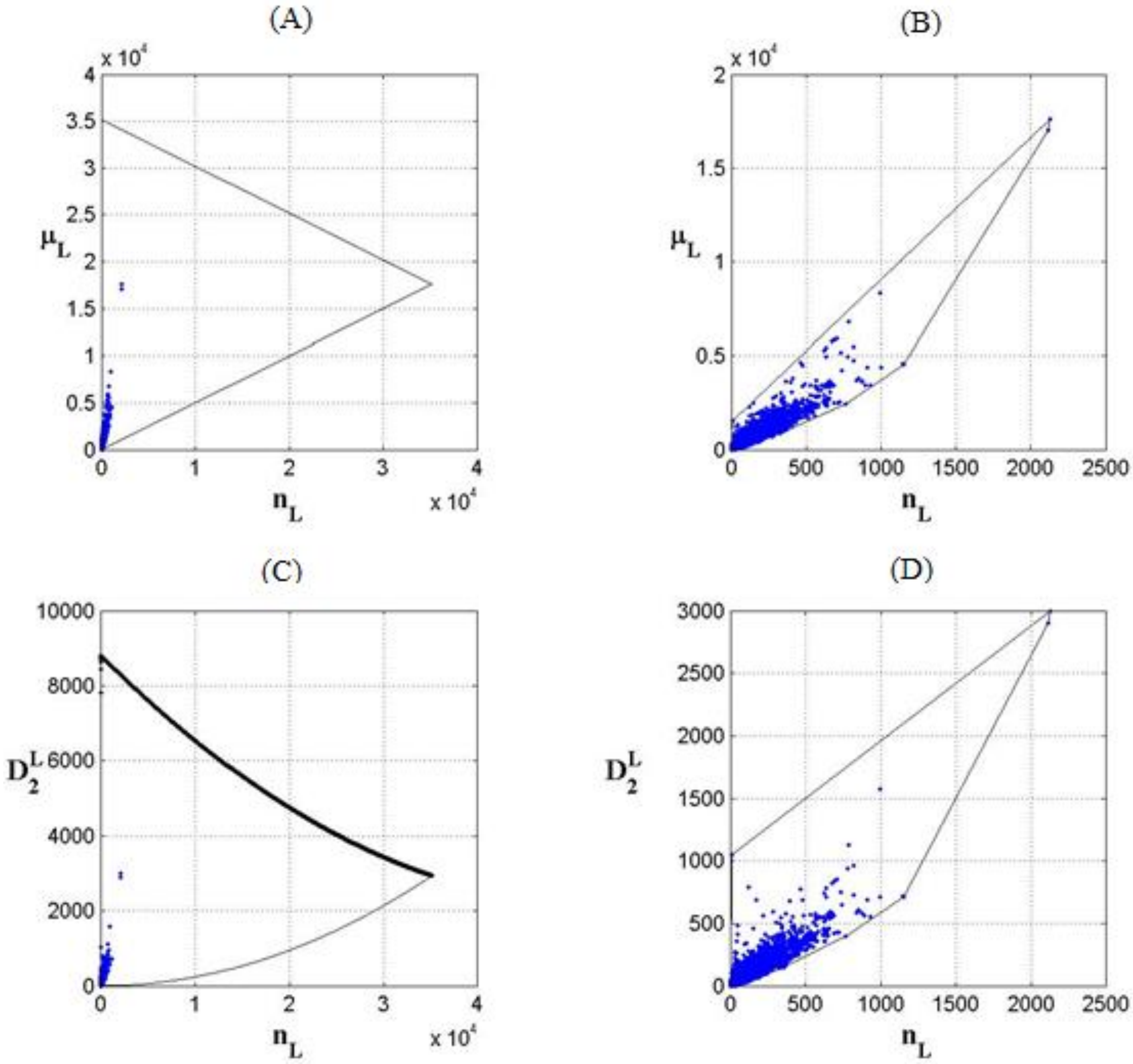

Figure 10: Protein Area Detail for Leucine

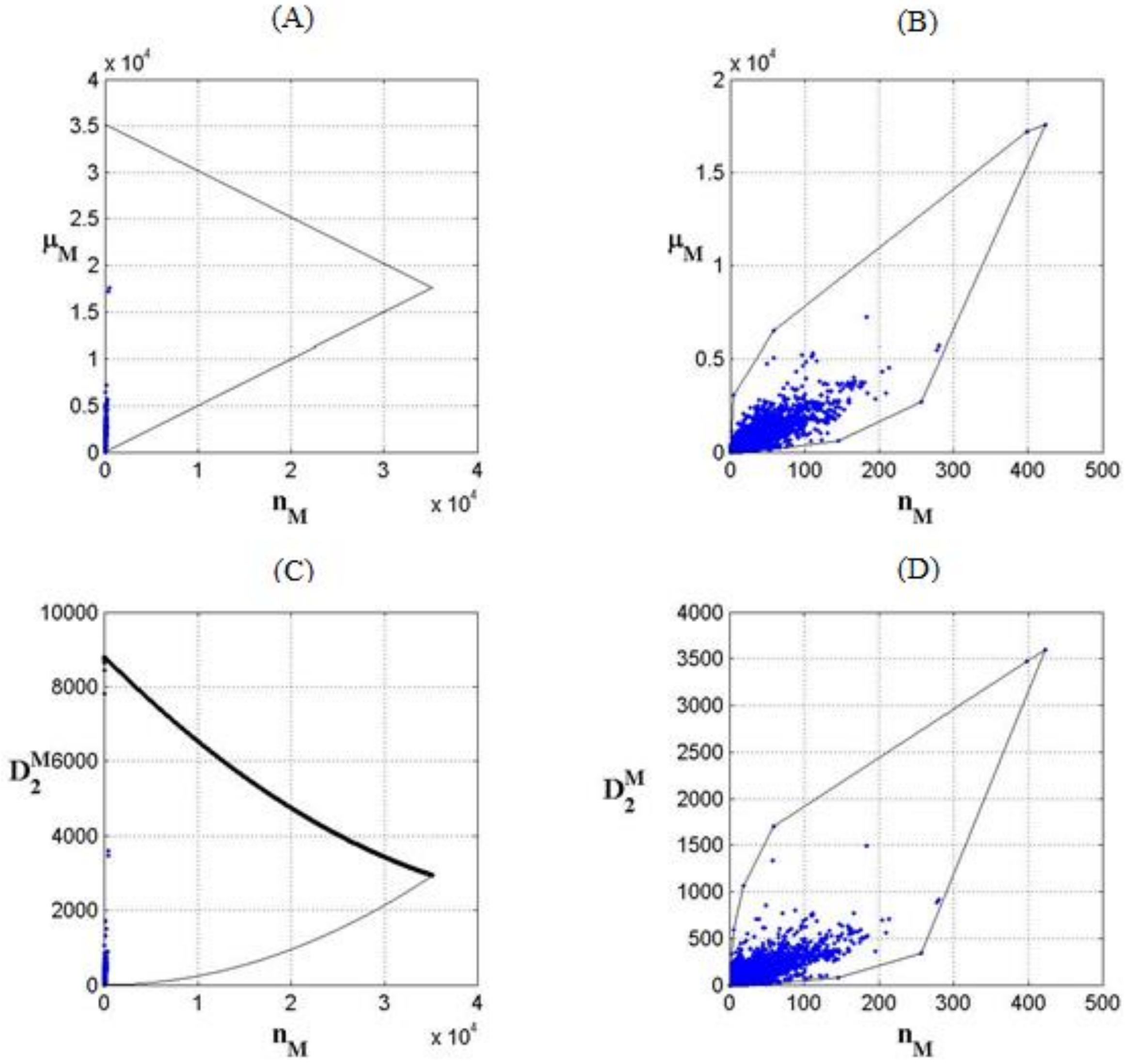

Figure 11: Protein Area Detail for Methionine

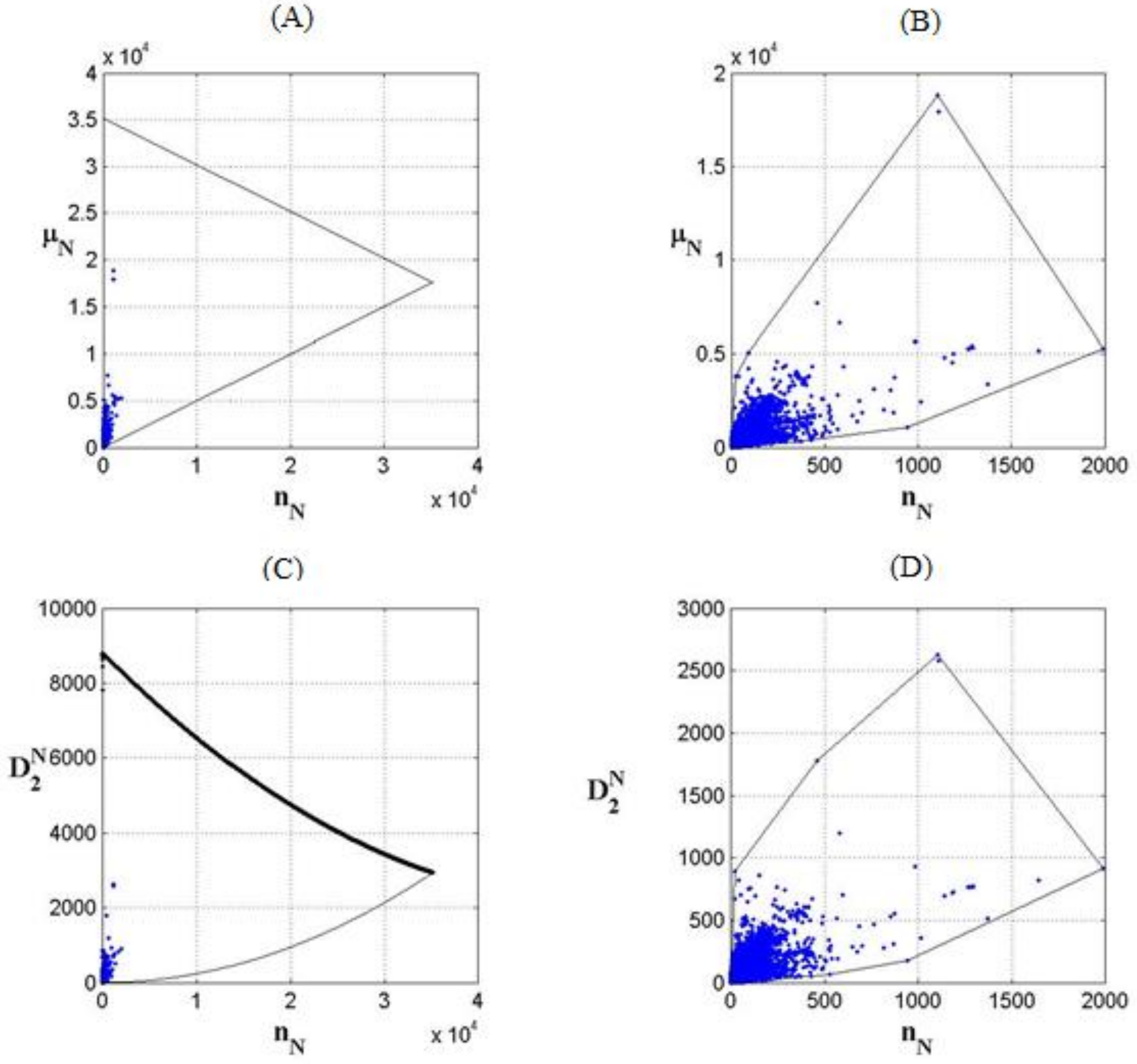

Figure 12: Protein Area Detail for Asparagine

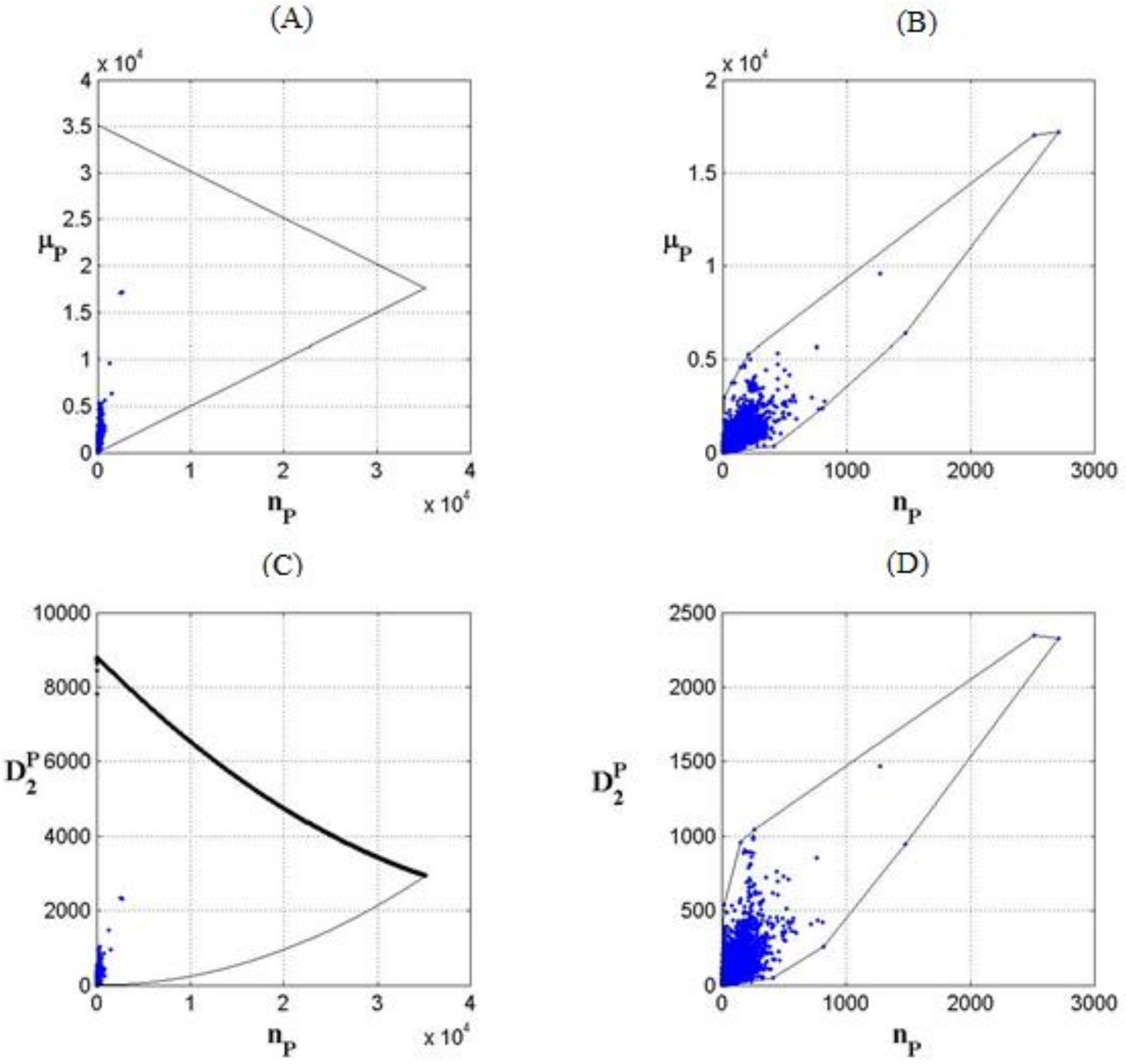

Figure 13: Protein Area Detail for Proline

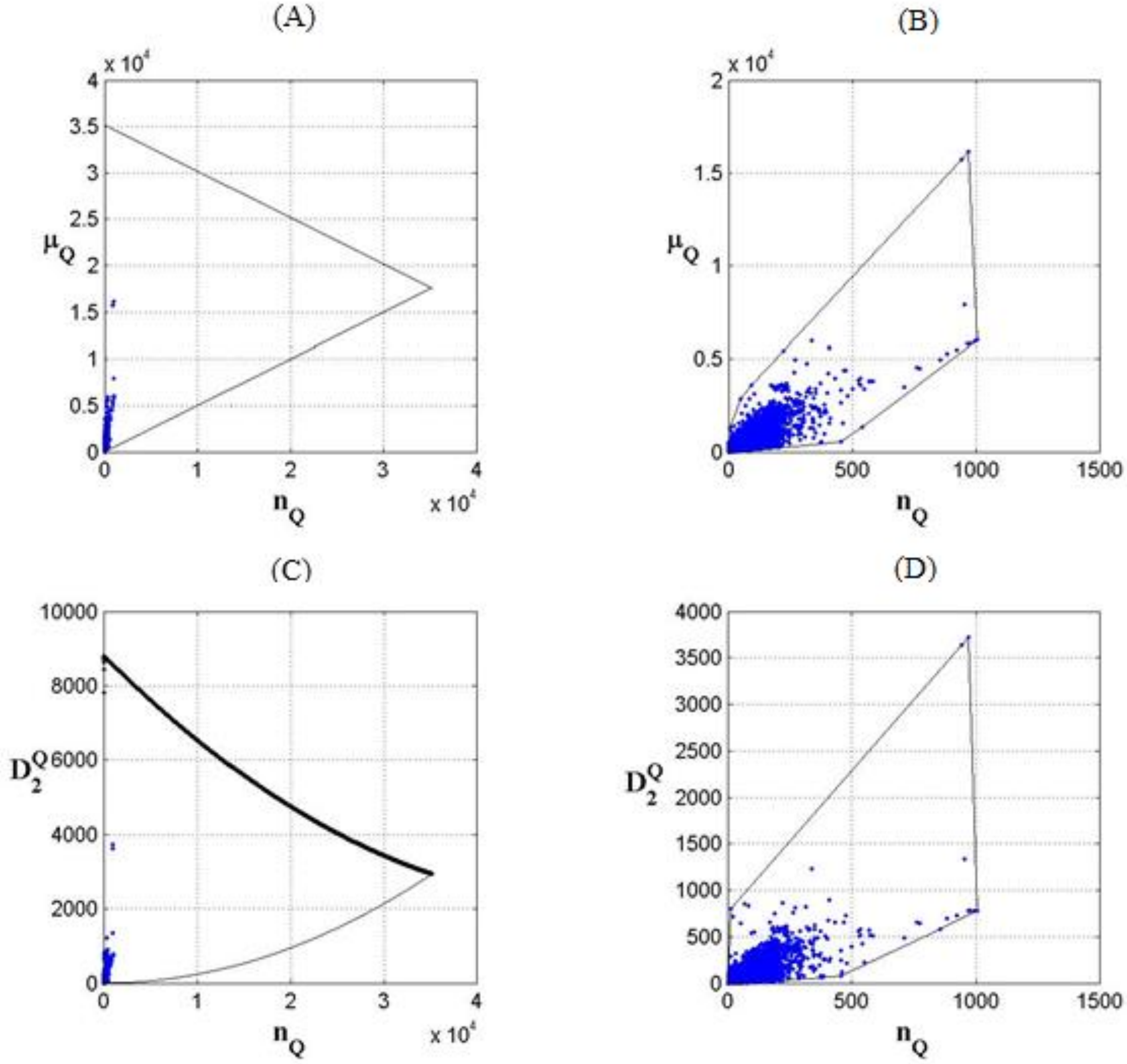

Figure 14: Protein Area Detail for Glutamine

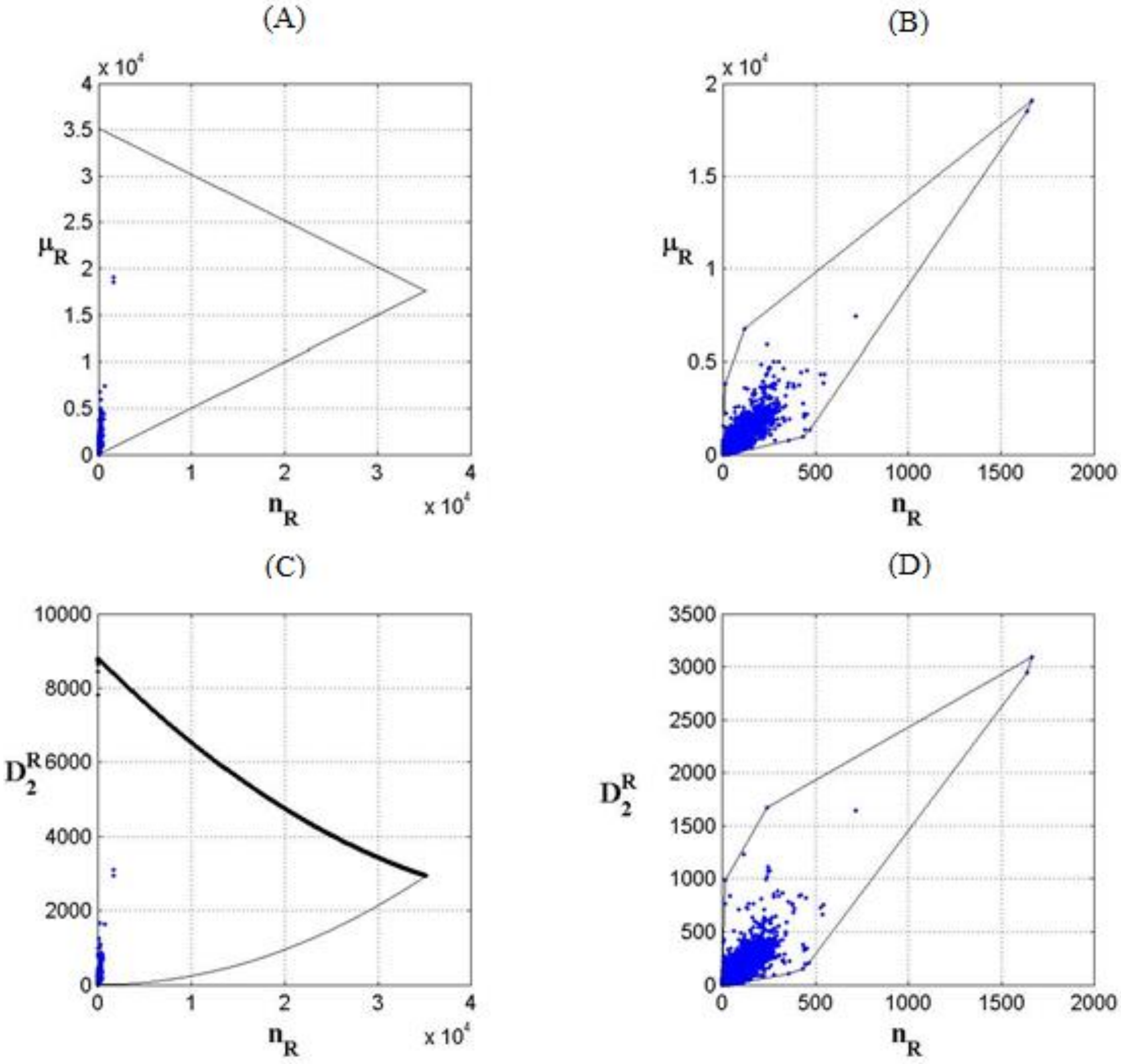

Figure 15: Protein Area Detail for Arginine

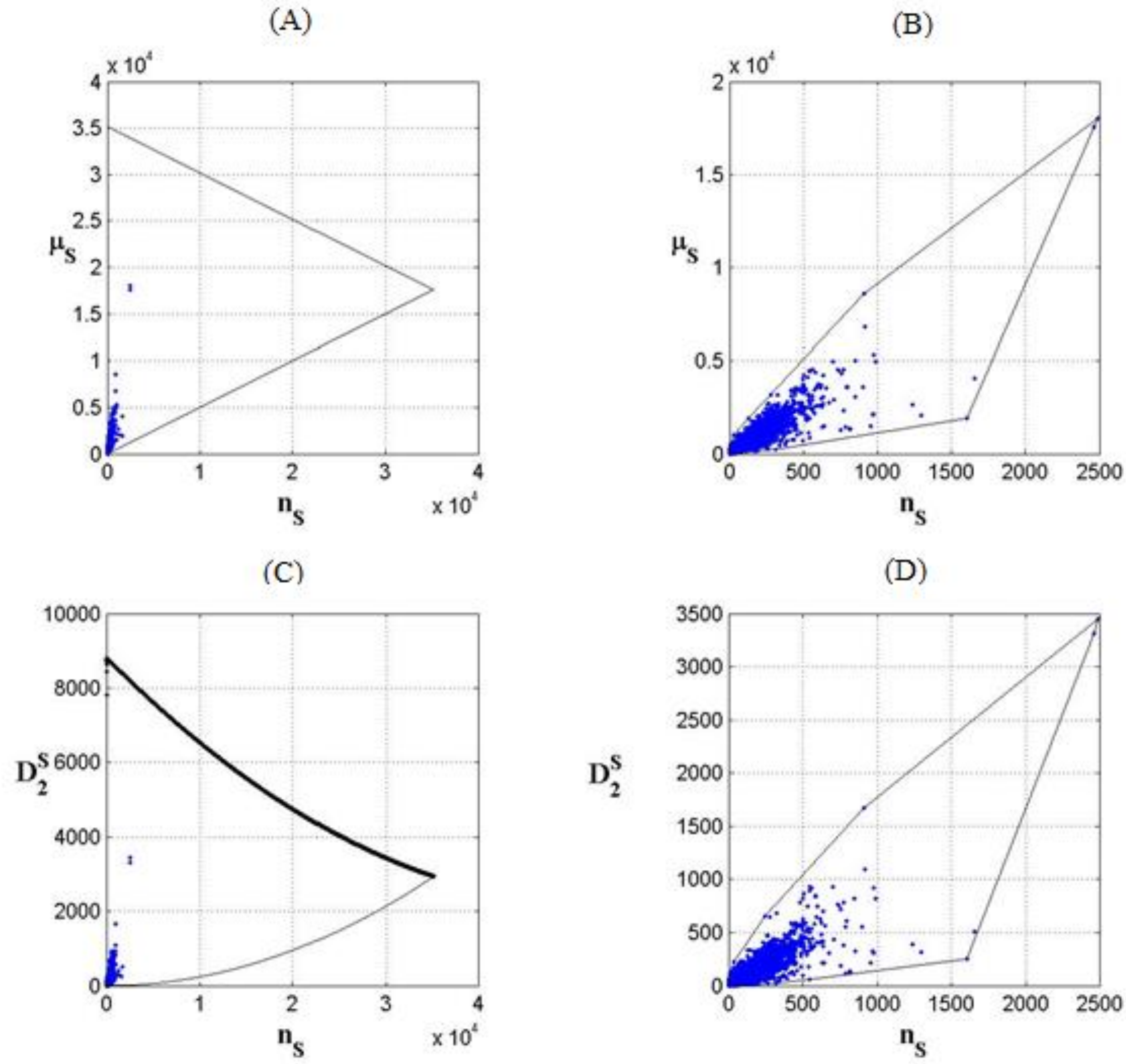

Figure 16: Protein Area Detail for Serine

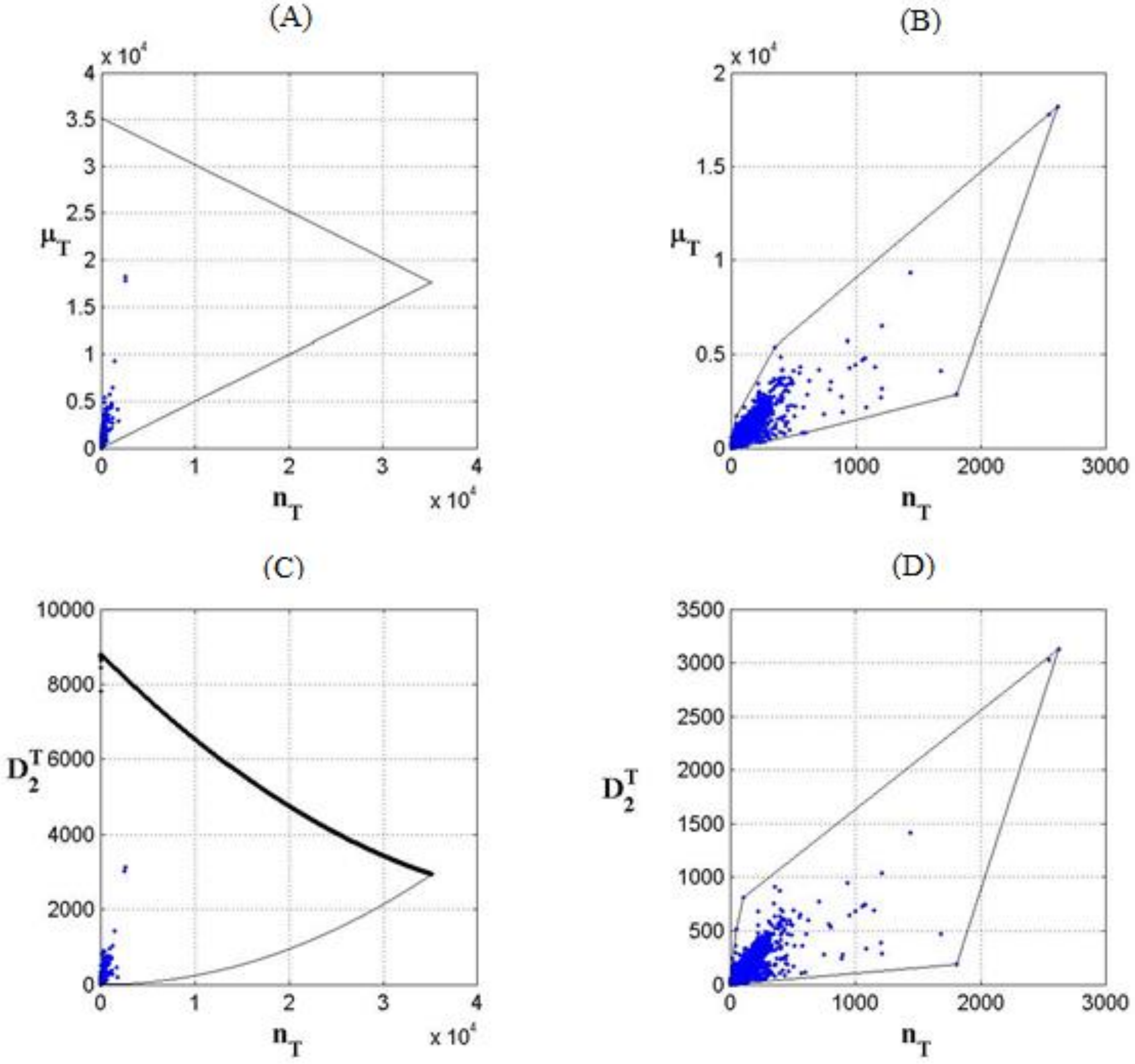

Figure 17: Protein Area Detail for Threonine

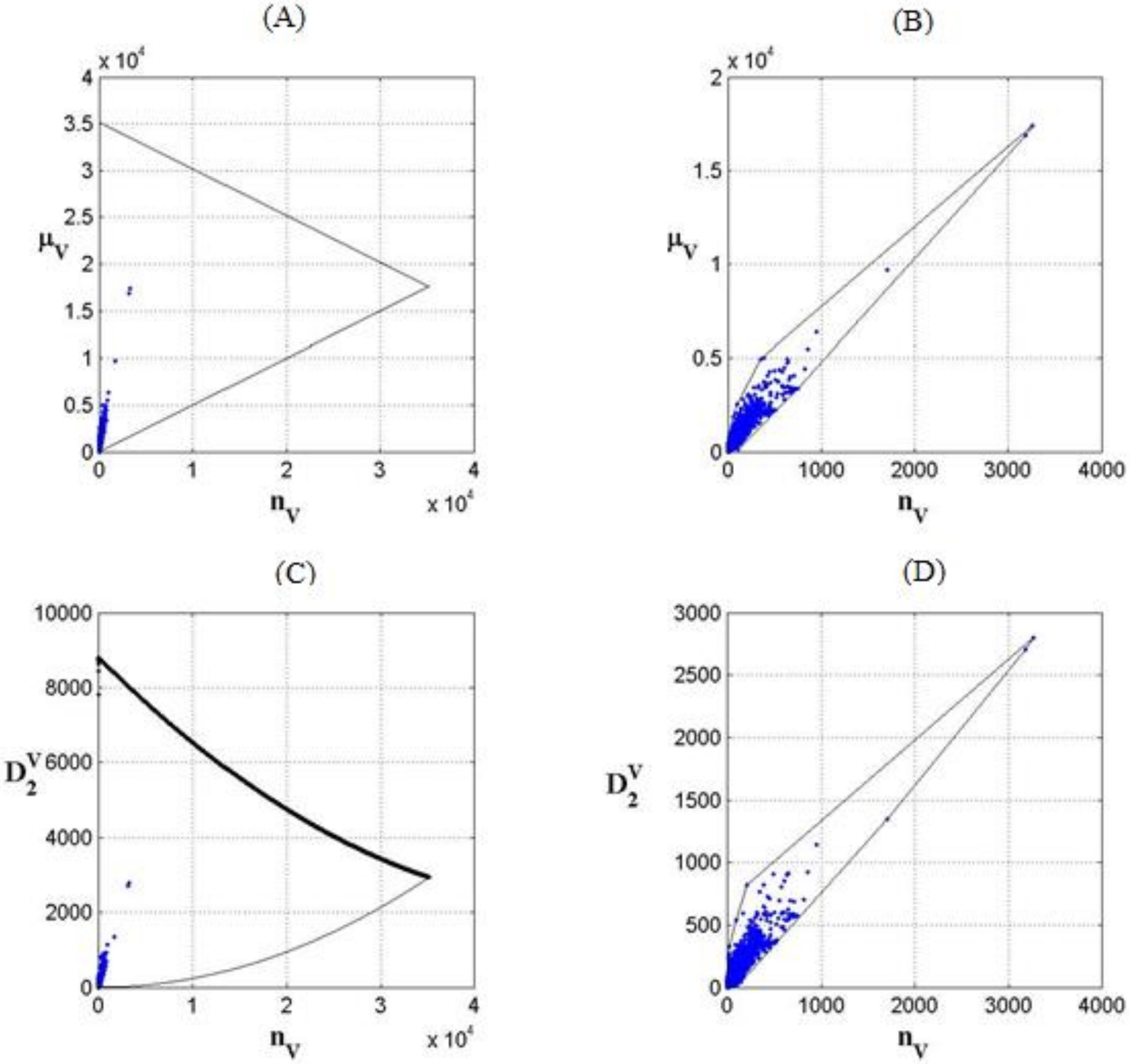

Figure 18: Protein Area Detail for Valine

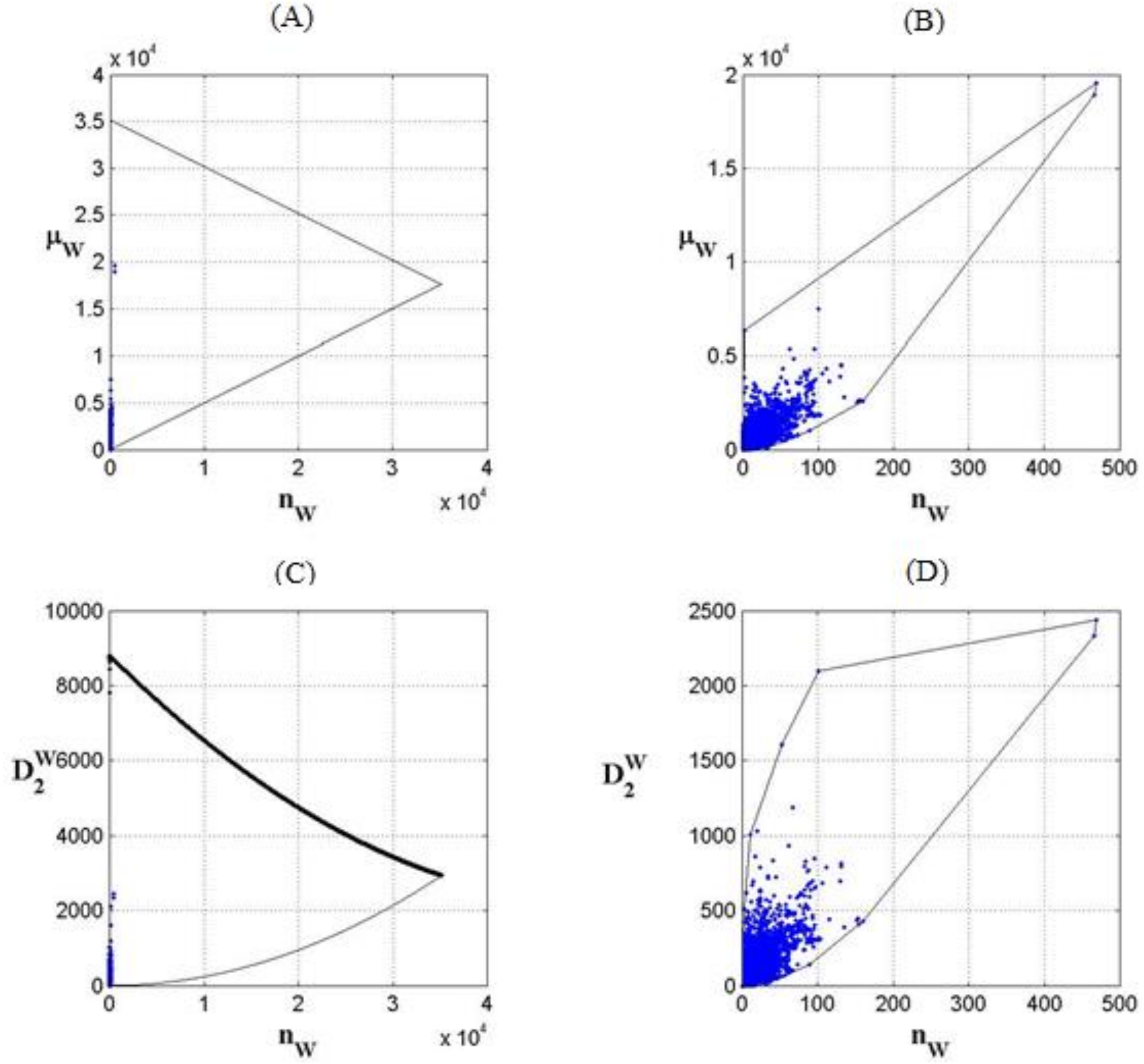

Figure 19: Protein Area Detail for Tryptophan

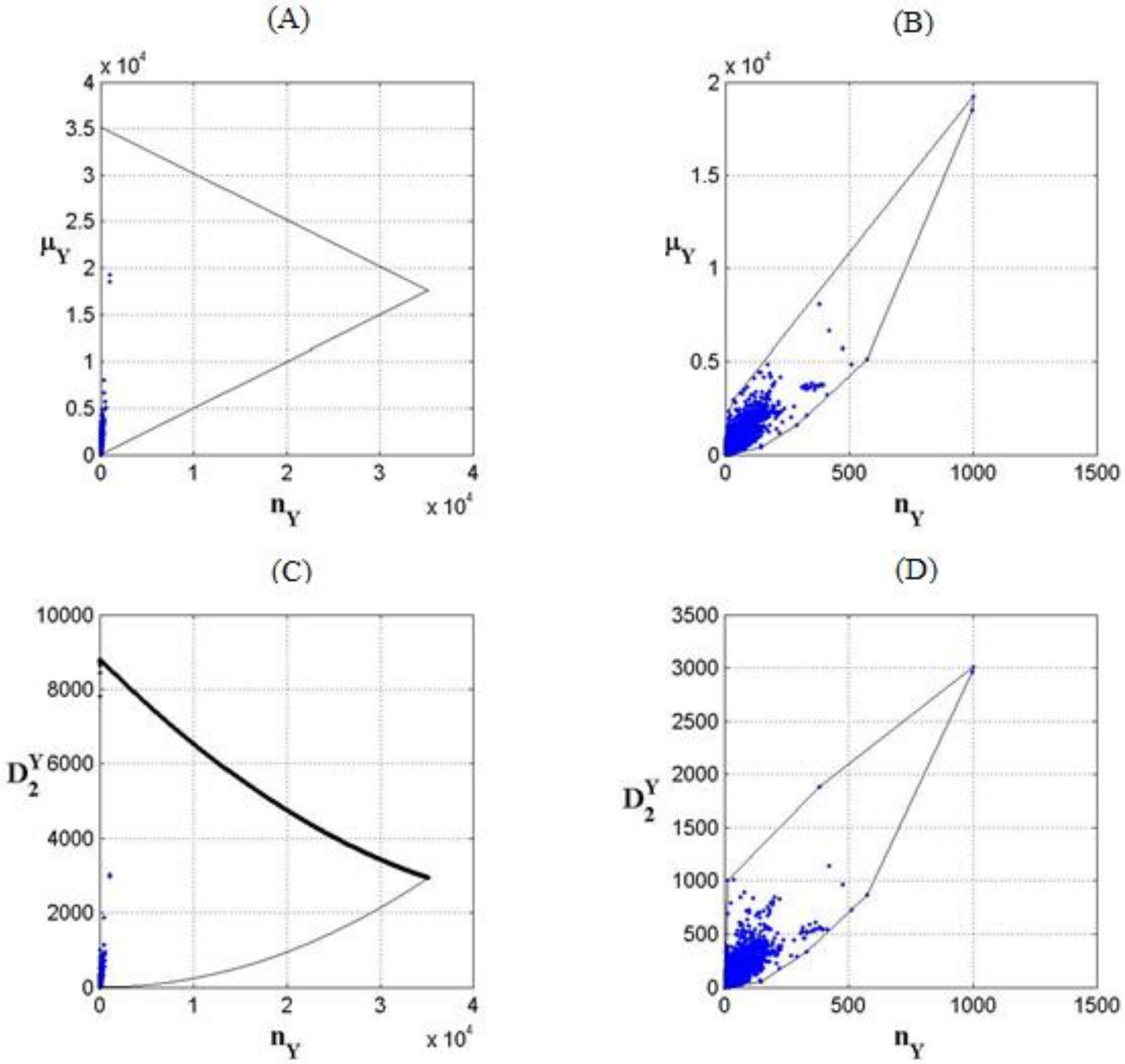

Figure 20: Protein Area Detail for Tyrosine
